# Supplementary material for: Psychometric Analysis and Contribution to the Evaluation of the Exams-Related Emotions Scale in Primary and Secondary School Students
Source: Int J Environ Res Public Health. 2022 Jun 1;19(11):6770. doi: 10.3390/ijerph19116770 (PMC9180762; doi:10.3390/ijerph19116770)
Supplement: Supplementary file 1 [file ijerph-19-06770-s001.zip › Cuestionario EEE.pdf]

UNIVERSIDAD DE BURGOS
Campus de Excelencia INTERNACIONAL

Emociones Relacionadas con los Exámenes (Mendo y León, 2020)

Hacer exámenes puede producir diferentes sentimientos. Este cuestionario hace referencia a las emociones que puedes experimentar cuando realizas exámenes.
Lee cuidadosamente y **Marca con una equis (X)** el **número** que indica la frecuencia con la que experimentas aquello que describe cada pregunta.

1	2	3	4	5
**NUNCA**	**CASI NUNCA**	**A VECES**	**CASI SIEMPRE**	**SIEMPRE**

Los siguientes ítems se refieren a los sentimientos que puedes experimentar **ANTES** de hacer un examen. Indica cómo te sientes generalmente antes de un examen.					
Antes del examen me siento nervioso e inquieto.	1	2	3	4	5
Me deprimo porque siento que no tengo mucha esperanza de aprobar el examen.	1	2	3	4	5
Pienso con optimismo en el examen.	1	2	3	4	5
No puedo ni pensar en lo vergonzoso que sería reprobar el examen.	1	2	3	4	5
Me preocupa si habré estudiado lo suficiente.	1	2	3	4	5
Como disfruto al preparar los exámenes estoy motivado para hacer más de lo necesario.	1	2	3	4	5
Estoy tan orgulloso de cómo me preparé que quiero comenzar el examen ya mismo.	1	2	3	4	5
Me pongo tan nervioso que desearía poder faltar al examen.	1	2	3	4	5
Preferiría no presentarme al examen porque he perdido todas las esperanzas.	1	2	3	4	5
Los siguientes ítems se refieren a los sentimientos que puedes experimentar **DURANTE** el examen. Indica cómo te sientes generalmente mientras haces el examen.					
Estoy muy seguro.	1	2	3	4	5
Me enojo.	1	2	3	4	5
Estoy muy nervioso.	1	2	3	4	5
Me siento avergonzado.	1	2	3	4	5
Estoy contento de poder afrontar el examen.	1	2	3	4	5
Creo que puedo estar orgulloso de mis conocimientos.	1	2	3	4	5
Me parece que las preguntas son injustas.	1	2	3	4	5
Me preocupa si aprobaré el examen.	1	2	3	4	5
Empiezo a pensar que por mucho que me esfuerce nunca aprobaré el examen.	1	2	3	4	5
Me da vergüenza lo mal que me preparé.	1	2	3	4	5
Como espero aprobar estoy motivado para esforzarme mucho.	1	2	3	4	5
Tengo ganas de abandonar.	1	2	3	4	5
Al comenzar el examen mi corazón empieza a acelerarse.	1	2	3	4	5
Me tiemblan las manos.	1	2	3	4	5
Me siento tan resignado que no tengo energía.	1	2	3	4	5
Los siguientes ítems se refieren a los sentimientos que puedes experimentar **DESPUÉS** de hacer el examen. Indica cómo te sientes generalmente después de hacer un examen.					
Estoy muy satisfecho conmigo mismo.	1	2	3	4	5
Me siento muy aliviado.	1	2	3	4	5
Siento vergüenza.	1	2	3	4	5
Me avergüenzan mis notas.	1	2	3	4	5
Cuando saco una mala nota quisiera no tener que volver a mirar a la cara a mi profesor.	1	2	3	4	5
Desearía poder retar al profesor.	1	2	3	4	5

Los nervios en el estómago desaparecen.	1	2	3	4	5
Estoy rebosante de entusiasmo.	1	2	3	4	5
Después del examen estoy tan orgulloso que me siento más grande.	1	2	3	4	5
Se me sube la sangre a la cabeza.	1	2	3	4	5
Por fin puedo volver a respirar tranquilo.	1	2	3	4	5
