# Supplementary material for: Psychometric Analysis and Contribution to the Evaluation of the Exams-Related Emotions Scale in Primary and Secondary School Students
Source: Int J Environ Res Public Health. 2022 Jun 1;19(11):6770. doi: 10.3390/ijerph19116770 (PMC9180762; doi:10.3390/ijerph19116770)
Supplement: Supplementary file 1 [file ijerph-19-06770-s001.zip › EES.pdf]

UNIVERSIDAD DE BURGOS
Campus de Excelencia INTERNACIONAL

Exams- related Emotions Scale (Mendo and León, 2020)

Doing tests can produce different feelings. This quiz refers to the emotions you may experience when you take exams.
Read carefully and Mark with an (X) the number that indicates how often you experience what each question describes.

1	2	3	4	5
**NEVER**	**ALMOST NEVER**	**SOMETIMES**	**ALMOST ALWAYS**	**ALWAYS**

The following items refer to the feelings you may experience **BEFORE** taking an exam. Indicates how you usually feel before an exam.					
Before the exam I feel nervous and restless.	1	2	3	4	5
I get depressed because I feel like I don't have much hope of passing the exam.	1	2	3	4	5
I think optimistically about the exam.	1	2	3	4	5
I can't even think of how embarrassing it would be to fail the exam.	1	2	3	4	5
I worry if I'll have studied enough.	1	2	3	4	5
As I enjoy preparing for exams I am motivated to do more than necessary.	1	2	3	4	5
I'm so proud of how I prepared that I want to start the exam right now..	1	2	3	4	5
I get so nervous that I wish I could miss the exam.	1	2	3	4	5
I would rather not take the exam because I have lost all hope.	1	2	3	4	5
The following items refer to the feelings you may experience **DURING** the exam. Indicates how you usually feel while taking the test.					
I'm very sure.	1	2	3	4	5
I get angry.	1	2	3	4	5
I'm very nervous.	1	2	3	4	5
I feel ashamed.	1	2	3	4	5
I'm happy to be able to take the exam.	1	2	3	4	5
I think I can be proud of my knowledge.	1	2	3	4	5
I think the questions are unfair.	1	2	3	4	5
I'm worried if I'll pass the exam.	1	2	3	4	5
I begin to think that no matter how hard I try, I will never pass the exam..	1	2	3	4	5
I'm embarrassed how badly I prepared.	1	2	3	4	5
As I hope to pass I am motivated to try hard.	1	2	3	4	5
I feel like quitting.	1	2	3	4	5
At the beginning of the exam my heart begins to accelerate.	1	2	3	4	5
My hands are shaking.	1	2	3	4	5
I feel so resigned that I have no energy.	1	2	3	4	5
The following items refer to the feelings you may experience **AFTER** taking the test. It indicates how you usually feel after taking an exam.					
I am very satisfied with myself.	1	2	3	4	5
I feel very relieved.	1	2	3	4	5
I feel ashamed.	1	2	3	4	5
I'm embarrassed by my notes.	1	2	3	4	5
When I get a bad grade I wish I didn't have to look my teacher in the face again .	1	2	3	4	5

When I get a bad grade I wish I didn't have to look my teacher in the face again .	1	2	3	4	5
Nerves in the stomach disappear.	1	2	3	4	5
I'm brimming with enthusiasm.	1	2	3	4	5
After the exam I am so proud that I feel bigger.	1	2	3	4	5
My blood goes up in my head.	1	2	3	4	5
Por fin puedo volver a respirar tranquilo.	1	2	3	4	5
